# Workers and Weariness

**Published:** 1893-09-09

**Authors:** 


### The Hospital

An Institutional Journal of

**Science, Medicine, Nursing, and Philanthropy.**

*For Hospitals, Asylums, Infirmaries, Dispensaries, Medical Practitioners, Students, Nurses, and the Charitable Public.*

Vol. XIV.—No. 363.] [Sept. 9, 1893.

### Workers and Weariness.

Professor Michael Foster has published his Rede lecture in the current number of the *Nineteenth Century*. Weariness is his subject, and it is one which demands the intelligent thought of all people of the present day. We recently called attention to the insanity which refuses to recognise weariness as the most dangerous symptom of mental fatigue. It way be true or false that the world will not see the year A.D. 2000. It is, however, undoubtedly true that the Mother of Parliaments, as the present House of Commons delights to call itself, is being rapidly reduced to a state of impotence which threatens the national safety. It is notorious that very old and very young people frequently have abnormal energy which spurs them on to acts of foolhardiness, which may, and frequently do, have a fatal termination. It would be impossible for any business undertaking of importance to be conducted successfully on the principles which at present govern the conduct of business in the House of Commons. Those principles as laid down by the Premier in the resolution of which he gave for Monday, and which were to apply to the rest of the present and the whole of the autumn session, could have but one result. In its modified form it is fatal to all useful legislation. That it was not strenuously opposed proves to demonstration the condition of weariness to which Members of Parliament have been brought. Any legislation pushed forward by the continuous use of the spur and the whip must prove defective and harmful to the wellbeing of the people. Members of Parliament being men are subject to physiological laws as all are, and the absence of proper rest, and the refusal to conduct business within reasonable hours can only result in breakdown and disaster.

The plan of business formulated by the Premier is opposed to every principle which enables any average mortal to master his work and maintain his health. In an ordinary business any such experiment as that about to be tried upon the House of Commons would very properly end in bankruptcy. We are no doubt a long way removed from national bankruptcy, but the Ship of State is now within measurable distance of irreparable damage and discredit. This is not a political journal, but common sense makes it apparent that the well-being of the people and the State demands the prompt presentation of a monster petition to the House of Lords not to pass the Appropriation Act, and to the Queen, urging the exercise of her prerogative, by the immediate dissolution of the present House of Commons.

Let us now see what Professor Foster has to say on the subject of weariness and its effects upon men and women. It is a popular belief that weariness is a physical symptom alone; that is to say, people become tired because their muscles are weary, whereas, in truth, muscular weariness depends not on the muscle alone, but on the manner in which the muscle in its work is aided and supported by the rest of the body. "The blood, sweeping throughout the whole body, washes out of the muscle all hurtful bodies, providing always the blood-stream is pure. If the blood-stream be sluggish, or if the blood coming to the muscle be already loaded with hurtful bodies, the clearance is slow or wholly fails, and weariness comes on apace. But even the simplest and rudest muscular tasks are not carried out by the muscles alone, for the brain and the nerves share in them too. It is a common experience that when we are weary almost, it may be, to death, some sudden emotion, some great joy or fear, may spur us to an effort which just before seemed impossible; conversely an emotion may appear to take from us all our muscular strength. Now the muscles neither know nor feel; their weariness cannot be affected by any emotion. That weariness which is put aside by hope, or which is hurried on by despair, must bea weariness not of the muscles, but of the nervous system." It would be interesting to follow Professor Foster as he goes on to prove the difference between the brain and the nerves, the brain being, of course, the central mechanism, the nerves mere bundles of fibres which carry the impulses to the muscles. We must, however, refer our readers to the article itself for this and other information, and would direct their attention especially to the experiments by which Professor Foster proves that the greater part at least of weariness is begotten not in the muscle, but in the brain.

Let us now consider a few of the mental symptoms of fatigue or weariness. Professor Foster insists that two facts must be grasped and remembered. Whilst it may be said of each member that the blood is the life thereof, it may with equal truth be said the blood is the death thereof; the blood is the channel for food, but it is also the pathway for poison. Again, all our knowledge goes to show that the work of the brain, like the work of the muscles, is accompanied by chemical changes, that the chemical changes, though differing in details, are of the same order in the brain as in the muscles. It is true that the changes in the brain are smaller than those of the muscle, but this is counterbalanced by the exceeding sensitiveness of the nervous substance; the last fact giving point to the caution that to do the maximum of brain work it is essential not to render the brain more agile, but to encourage its humble helpmates so that their more efficient co-operation may defer the onset of weariness.

Mind and body being thus intimately associated, no sensible person will risk the destruction of health by neglecting either the one or the other. Sleep, good food, healthy surroundings, rational hours of work, and sufficient exercise are points which many people have long recognised as essential to their well-being, yet the race for wealth, or for political advancement, or the mere struggle for existence, may cause the most enlightened to neglect them. At first a man so circumstanced attributes his lassitude, which is usually confined to the early morning, to anything but the right cause—a feeling of depression and a sense of ill-being may make him miserable, but unless his doctor takes him in hand they will not check his downward course. Next he will become oppressed with the need of increased effort, a loss of memory, and the difficulties of remembering what he reads. To this point a man may advance without fatal injury, but should he proceed until everything appears to him dark and hopeless, a worry and an apprehension, then he may be beyond rescue, and his next step may land him in a lunatic asylum if it does not terminate earlier in a graveyard.

Dr. Cowles, of the McLean Hospital, has written a most interesting paper on the mental symptoms of fatigue, which all brain workers should read. He shows in a practical way how difficult it is to restore the weary to energy and strength. Overworked women, professional men, politicians, and others "work on their nerves," and say they "don't feel tired, and nothing is the matter." Many of them, indeed, insist that they feel better when actively pursuing their ordinary occupations. Dr. Cowles declares this condition, which comes on insidiously, to be a most dangerous one. With the impairment of the natural fatigue sense the mental effect is that a man will not believe even his physician's diagnosis of fatigue. He is, therefore, prone to look for some other reason for his sense of ill-being and inefficiency, and finds in retrospection cause for self-reproach and hopelessness in the future, or insists upon a revolution in his affairs as the only remedy for a condition of which he himself is the central cause. Here, then, we arrive at the two great factors which have to be faced to-day in our national and political life. The first, or retrospective cause, lies at the root of the present epidemic of suicide of which the papers are full. The second, or impairment of judgment cause, lies at the root of the political *impasse*, which its authors declare can be cured only by revolution. The papers of Professor Michael Foster and Dr. Cowles are therefore most timely, and if the lessons they teach can be driven home they may prove fruitful by producing a more natural and healthy tone not only in our Parliamentary but in national life too.

